# How do we deliver our findings? Analysis of podium presentations at shoulder meetings

**DOI:** 10.1186/s13018-018-0942-7

**Published:** 2018-09-14

**Authors:** Joan Miquel, Fernando Santana, Albert Barrera, Carlos Torrens

**Affiliations:** 1 Orthopaedics and Trauma Department, Consorci Sanitari de l’Anoia, Avda de Catalunya, 11, 08700 Igualada, Barcelona Spain; 2 Orthopaedics and Trauma Department, Parc de Salut Mar, Passeig Marítim, 25-29, 08003 Barcelona, Spain

**Keywords:** Shoulder congresses, Time constraints, Video inclusion, Conference presentations

## Abstract

**Background:**

The aim of the present study was to evaluate the time structure of oral presentations delivered at three shoulder congresses: shoulder sessions at the American Academy of Orthopaedic Surgeons (AAOS) Meeting, European Foundation of National Associations of Orthopaedics and Traumatology (EFORT) Congress, and International Congress of Shoulder and Elbow Surgery (ICSES).

**Methods:**

A total of 160 oral presentations at the 2016 AAOS Annual Meeting, 17th EFORT Congress, and 13th ICSES were included. Podium presentations were categorized by topic, congress, inclusion of video support, and nationality of the speaker. Total time and time dedicated to each section of the presentation (introduction, methods, results, discussion and conclusions) were collected for all podium presentations.

**Results:**

Approximately 34% of speakers exceeded time constraints. No differences were found in the times that presenters used for the introduction, methods, results, and conclusions sections (*p* > 0.05). However, when extended introductions were delivered, the results and conclusions sections were shortened (*r* = − 0.2 and *r* = − 0.21, respectively). Inclusion of video support tended to result in exceedance of time limits (*p* < 0.01).

**Conclusions:**

One third of the shoulder surgeons exceeded time constraints in their conference presentations, and no distinctions were found in time allocations for different sections of the presentations. Longer introductions may lead to time restriction in the results and conclusions sections.

## Background

Presentations at medical congresses are the fastest way to spread scientific data across the community. Hundreds of oral presentations are given annually at orthopedic forums, and some guidelines exist in the medical literature regarding the elements needed to deliver an effective scientific presentation [1–9]. These recommendations are frequently based on expert opinions [5–8] or are focused on the technical aspects of the presentation [9, 10].

When the allotted time for oral presentations at congresses is limited, authors must present a high volume of data in a short period of time, emphasizing the most relevant findings. Presentations that exceed time constraints seem to be unfavorably received by the audience [6], and experts recommend avoiding overly detailed slides and spending no more than 1 min on each slide [5]. Authors may concentrate on the methods and results sections, as they represent the main contributions of their study. Moreover, concisely presented background information and key conclusions are associated with higher overall presentation quality [7].

Various instruments have been created to objectively evaluate the content and style of presentations [7, 8], but little information is available on time distributions in the delivery of scientific presentations.

## Methods

The aim of the present study was to evaluate the time structure of oral presentations in shoulder and elbow sessions at three outstanding orthopedic congresses.

A total of 160 oral presentations given at the 2016 American Academy of Orthopaedic Surgeons (AAOS) Meeting, 2016 European Foundation of National Associations of Orthopaedics and Traumatology (EFORT) Congress, and 2016 International Congress of Shoulder and Elbow Surgery (ICSES) were included. Oral presentations were classified by topic (basic science, proximal humeral fractures, cuff disorders and treatment, shoulder arthroplasties, shoulder instability, clavicle fractures and acromioclavicular joint diseases, biceps pathology and frozen shoulder, and miscellaneous) and by congress. Only podium presentations corresponding to original studies were included in this study. Instructional courses, symposiums, and invited lectures were excluded for the purpose of this analysis.

A single observer timed the four sections (introduction, methods, results, discussion and conclusions) of all podium presentations using a digital chronometer (iPhone 5, Apple iOs, version 9.3). Evaluations of video inclusions in the presentation were recorded for all presentations at ICSES. Therefore, 56 presentations were available to study the influence of video support on presentation time distribution.

Each section time was weighted based on the total time allotted in each congress to compare all examined presentations: a total of 5 min for EFORT and ICSES and 6 min for AAOS. Then, percentage of time was used to compare presentations across congresses.

### Statistical analysis

Pearson’s correlation coefficient was calculated for each pair of variables, and the corresponding 95% confidence intervals were computed to analyze the relation between the time dedicated to the introduction, remaining sections of the presentation (methods, results, and conclusions), and total presentation time. In addition, the proportion of presentations exceeding 5 min at ICSES that included video support and the proportion of those without video inclusion were compared with a Chi-squared test.

Correlations between different sections of the podium presentations were represented by the correlation coefficient (*r*) and its standard deviation. Differences were considered significant at *p* < 0.05. Statistical analysis was performed using SPSS 18.0 (SPSS Inc., Chicago, IL).

## Results

Seventy-two presentations (45%) from the AAOS Meeting, 56 from the ICSES (35%), and 32 from the EFORT Congress (20%) were studied. The distribution of studied presentations per topic is shown in Table 1. All speakers were categorized as Doctor of Medicine (MD). Speakers allotted similar times to the introduction, methods, results, and conclusions sections without significant differences (25.4% to the introduction, 26.5% to the methods, 27.7% to the results, and 21.4% to the conclusions, *p* = 0.537).

**Table 1:** Distribution of oral presentations regarding the topic of the conference

Topic	NUM presentations	% Presentations
Basic science	6	3.8
Proximal humeral fractures	35	21.9
Cuff disorders	29	18.1
Shoulder arthroplasties	64	40
Shoulder instability	17	10.6
Clavicle fractures and AC joint disorders	12	9.5
Biceps pathology and frozen shoulder	15	9.4
Miscellaneous	11	6.9

**Fig. 1:**
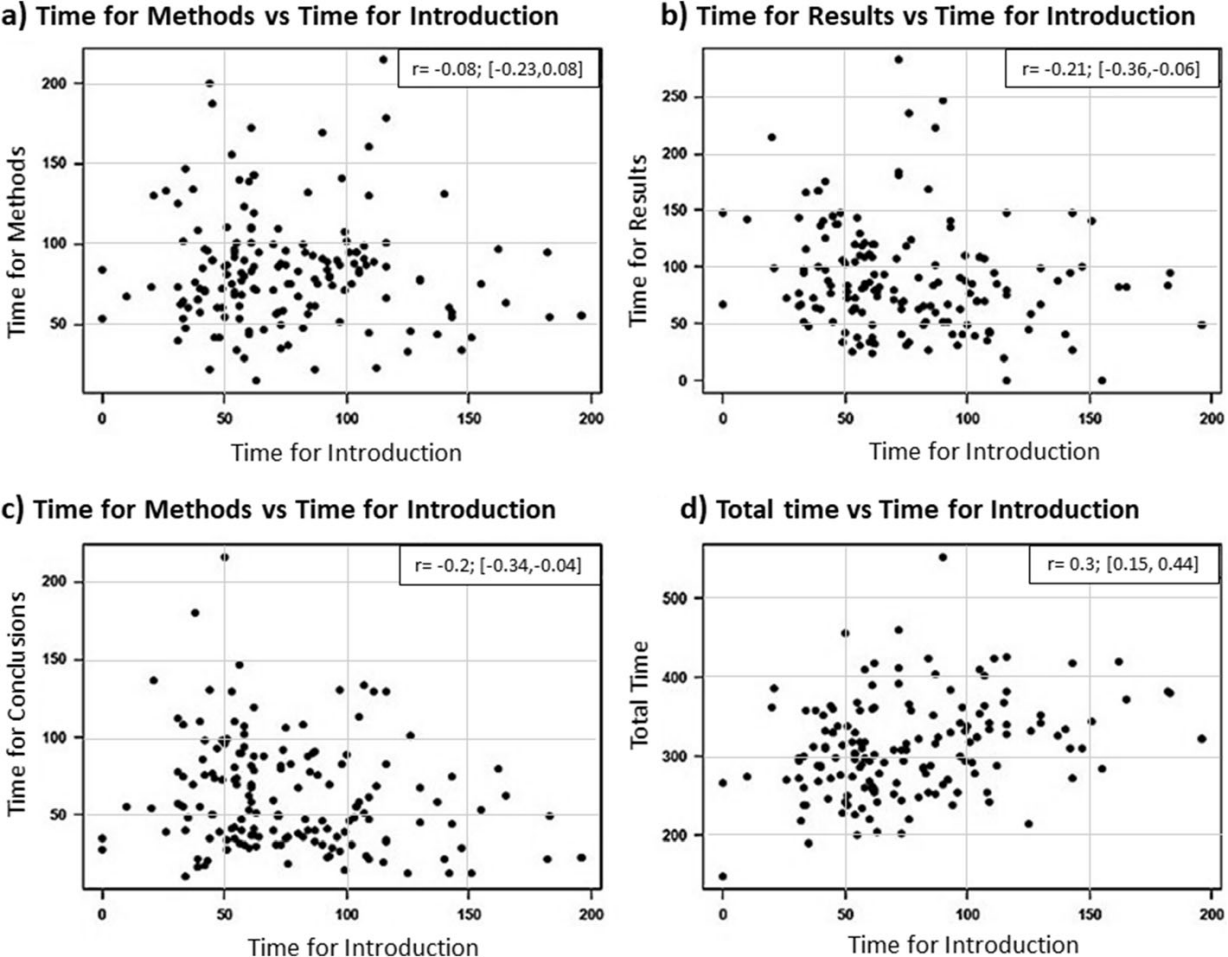
Correlation between time dedicated to the introduction section and the rest of the paper sections. **a** Correlation between the introduction and methods. **b** Correlation between the introduction and results. **c** Correlation between the introduction and conclusions. **d** Correlation between the introduction and total time for presentation

Fifty-four of the 160 presentations (33.8%) exceeded the time constraint stipulated by the congress organization. The time distributions for all sections of the podium presentations itemized by congress are shown in Table 2. The authors who delivered longer introductions had shortened results and conclusions sections (*r* = − 0.2 and *r* = − 0.21, respectively) (Fig. 1). Consequently, the time dedicated to the introduction section did not predispose presenters to exceed the time limit (*p* = 0.423), nor did the time allocated to the methods (*p* = 0.918), results (*p* = 0.301), or conclusions (*p* = 0.375) sections. Twelve of the 56 ICSES presentations included videos. Of these 12 presentations, 10 exceeded the time constraint allotted by the congress organization. In contrast, only 13 out of 44 speakers exceeded the time allocation when video support (*p* = 0.001) was not included in the presentation. Conference topic did not affect time duration (*p* = 0.591).

**Table 2:** Time distribution for oral presentation according to different congress

Congress	AAOS	EFORT	ICSES	*p* value
Number present	72	32	56	
Mean total time (s)	331.79 (214–552)	284.34 (199–425)	298.02 (147–460)	
INTRO				
Seconds	80.90 (10–196)	71.09 (31–137)	79.39 (0–385)	0.57
Percentage	24.53 (3.65–61.06)	25.59 (10.58–45.04)	26.08 (0–100)	
Methods				
Seconds	92.57 (33–214)	75.35 (22–200)	74.79 (0–178)	0.44
Percentage	28.00 (10.11–58.15)	26.22 (8.76–55.10)	25.55 (0–54.20)	
Results				
Seconds	97.00 (20–247)	74.28 (31–181)	85.27 (0–282)	0.41
Percentage	29.07 (5.43–64.21)	25.74 (12.25–53.01)	28.43 (0–61.30)	
CONCL				
Seconds	61.32 (10–180)	63.84 (17–130)	58.57 (0–216)	0.14
Percentage	18.39 (2.80–50.28)	22.43 (5.12–38.23)	19.93 (0–51.05)	
# presentations with extended time	20 (37%)	11 (20.4)	23 (42.6%)	0.28

*AAOS* American Academy of Orthopaedic Surgeons, *EFORT* European Foundation of National Associations of Orthopaedics and Traumatology, *ICSES* International Congress of Shoulder and Elbow Surgery, *INTRO* introduction, *CONCL* conclusions

## Discussion

Podium presentations are a challenge for investigators as they require the synthesis of an entire research project into a short (5- to 7-min) presentation. No specific training on presentations is commonly offered to speakers, and few guidelines exist in the literature regarding the art of presenting [1–10]. Commonly, these recommendations are based on the expert author’s personal guidelines [5, 6]. This study found that 30% of speakers exceeded the time constraints allotted by the congress organization. In addition, speakers delivered unbalanced presentations by allocating the same amount of time to various sections with different impacts on the audience’s understanding of the paper as a whole.

Presentations that meet time constraints are more positively received by audiences. Based on the results of our study, more than 30% of the studied presentations would have been unfavorably received by the audience in terms of their time durations. Moreover, these data can be useful for subsequent shoulder surgery congress committees to incorporate free paper sessions within scheduled times.

The time distributions of the studied conferences merit further discussion. As stated by Waljee et al. [7], in a typical scientific presentation, the methods and results sections often comprise most of the allotted time as they require clear discussion. However, a common error is including several background slides, which limits the time available for the results section [10]. Our findings support this statement, as the speakers used the same amount of time for each section without discriminating between sections. However, the speakers who delivered longer introductions tended to shorten their final sections (results and conclusions) to accommodate time constraints, which could impact their overall presentation quality. Based on our data, excessive background information and slide content are not recommended as they may cause the speaker to accelerate essential parts of the presentation. A clear and concise display of background information is adequate and is significantly associated with higher presentation quality [7].

One in four authors included video support in their presentation as an illustration or to show a specific surgical technique. Video inclusion may be helpful for orientating the audience and enhancing overall presentation quality. However, video support may lengthen presentation times and may lead exceedance of the time limits allocated by the congress organization.

Few guidelines exist to objectively evaluate the value of a congress presentation. Farrokhyar and colleagues [8] developed an instrument to assess scientific podium presentation quality for a 10-min presentation. This instrument grants a maximum of 55 points for a perfectly completed presentation: 20 marks for scientific content, 30 marks for style/skills, and 5 marks for overall impression. Among these 55 points, only the final 5 points are allocated to marks for the presenter’s overall preparation and presentation skill within the allotted time. Moreover, the total score of each presentation is weighted to 100% as a final mark, giving a twofold higher scoring weight to scientific content than to the style/skills of the presenter. Furthermore, time adaptation of the presentation is poorly represented in this instrument, and it may overrate presentations that exceed time constraints. A different system to evaluate presentations based on the development of a scoring rubric was used to evaluate resident research oral presentations. This system also does not grant specific points to presentation time constraints [11]. The awareness of the importance of the medical presentation dexterity is progressively rising in medical education arena. Skills in oral presentation represent a critical factor in current educational trends for medical students [12, 13].

Some recommendations can be made based on the results of this study. First, a presentation that is balanced with respect to the relevance of different sections is important. Presenter effort and time expenditure should be focused on the methods and results sections, as they represent the main contributions of the study. Limiting the time dedicated to the introduction and conclusion sections may be helpful for adapting a presentation to time constraints. Second, a 30% rate of presentations exceeding time constraints may threaten the time allocated for questions from both the moderators and audience; this time period is important as it represents a unique opportunity to discuss scientific projects with the authors. Congress organizing committees can address these issues by offering specific guidelines in podium acceptance notifications (as they often do for poster presentations).

This study has several limitations. First, audience perception of the presentations was not examined. The findings of this study cannot be correlated to audience impressions. Second, the impact of video support on time length was studied at only one congress. However, the findings presented in this paper may improve our understanding of these aspects of presentations and can be used to both streamline speaker preparation and enhance the communication of key research findings. Finally, no specific speakers’ subcategorization (academic teacher, fellow, resident) has been performed for this study. In consequence, the effect of speaker expertise on podium time distribution remains unknown.

## Conclusions

More than 30% of shoulder congress presenters exceeded time constraints at conferences, and the presenters made no distinctions when allocating time to different sections of their presentations. Longer introductions may lead to time restrictions in the results and conclusions sections.

## Abbreviations

AAOS: American Academy of Orthopaedic Surgeons
EFORT: European Foundation of National Associations of Orthopaedics and Traumatology
ICSES: International Congress of Shoulder and Elbow Surgery
